# Image quality evaluation of intra‐irradiation cone‐beam computed tomography acquired during one‐ and two‐arc prostate volumetric‐modulated arc therapy delivery: A phantom study

**DOI:** 10.1002/acm2.13095

**Published:** 2020-11-16

**Authors:** Hiraku Iramina, Ayaka Kitamura, Mitsuhiro Nakamura, Takashi Mizowaki

**Affiliations:** ^1^ Department of Radiation Oncology and Image‐applied Therapy Kyoto University Hospital Kyoto Japan; ^2^ Division of Medical Physics Department of Information Technology and Medical Engineering Human Health Sciences Graduate School of Medicine Kyoto University Kyoto Japan; ^3^ Department of Radiation Oncology and Image‐applied Therapy Graduate School of Medicine Kyoto University Kyoto Japan

**Keywords:** intra‐irradiation CBCT, MV‐scatter, VMAT delivery

## Abstract

**Purpose:**

To evaluate (a) the effects of megavoltage (MV)‐scatter on concurrent kilovoltage (kV) projections (*P*_MVkV_) acquired during rotational delivery, and (b) the image quality of intra‐irradiation cone‐beam computed tomography (ii‐CBCT) images acquired during prostate volumetric‐modulated arc therapy (VMAT) delivery.

**Methods:**

Experiment (1): *P*_MVkV_s were acquired with various MV beam parameters using a cylindrical phantom: field size (FS), MV energy (6 or 15 MV), dose rate (DR), and gantry speed. The average pixel values were calculated in a region on each *P*_MVkV_ which were extracted at eight equally spaced gantry angles. Experiment (2): 11 one‐arc and seven two‐arc 15 MV prostate VMAT plans were used along with a pelvis phantom. One plan was selected from each of arc plans and its MV energy was changed to 6 MV. After *P*_MVkV_s were acquired, projections consisting of MV‐scatter only (*P*_MVS_) were acquired with closing kV blades and subtracted from *P*_MVkV_ (*P*_MVScorr_). Projections by kV beams only were acquired (*P*_kV_). The corresponding CBCT images were reconstructed (CBCT_MVkV_, CBCT_MVScorr_, and CBCT_kV_). The root‐mean‐square errors (RMSEs) were calculated in prostate region and 3D gamma analysis was conducted, in which the CBCT‐number was used instead of doses between ii‐CBCT images and CBCT_kV_ (30 HU/1 mm).

**Results:**

Experiment (1): The MV‐scatters were dependent on the FSs, MV energies, and DRs. Experiment (2): The median RMSEs for CBCT_MVScorr_ were decreased by 107.5 HU (1‐arc) and 42.9 HU (2‐arc) compared to those for CBCT_MVkV_. The median GPRs for CBCT_MVScorr_ were 94.7% (1‐arc) and 93.4% (2‐arc), while those for CBCT_MVkV_ were 61.1% and 79.9%, respectively. GPRs for 6 MV plans were smaller than those for 15 MV plans.

**Conclusions:**

The number of MV‐scatters increased with larger FSs and DRs, and smaller MV energy. The MV‐scatters were corrected on the CBCT_MVScorr_ regardless of the number of arcs.

## 1 INTRODUCTION

Image‐guided radiotherapy has been developed extensively in the past 2 decades.^1^ One such method is cone‐beam computed tomography (CBCT) image acquisition by a linear accelerator (linac)‐mounted kilovoltage (kV) imaging subsystem. With the advent of CBCT, it has become possible to confirm the location of internal organs in the treatment position prior to megavoltage (MV) beam irradiation.^2^

Subsequently, the demand for the monitoring of the target or internal organs during MV beam irradiation has increased. Poulsen et al. proposed kilovoltage intrafraction monitoring (KIM), which is a three‐dimensional (3D) target position estimation method during MV beam irradiation for the prostate region.^3^, ^4^, ^5^ The authors estimated the 3D positions of implanted radiopaque markers with a monoscopic view by using spatial probability density. KIM has been used for the prostate and liver with volumetric‐modulated arc therapy (VMAT).^6^, ^7^, ^8^, ^9^, ^10^ However, KIM only extracts 3D point positions and cannot generate 3D volume images.

In routine clinical practice, it is desirable to obtain 3D images of the actual delivered dose distributions for adaptive radiotherapy and accurate prognosis prediction. To calculate the distributions, 3D volume images should be acquired during MV beam irradiation, which can reflect the actual positions of the target or internal organs inside the patient body. Although CBCT images that are established prior to MV beam irradiation can be used for this purpose, the internal organ positions may differ during the setup and irradiation. CBCT acquisition methods during rotational therapy such as VMAT delivery, or intra‐irradiation CBCT (ii‐CBCT) acquisition, have been presented to acquire 3D volume images during MV beam irradiation.^11^, ^12^, ^13^, ^14^ A major issue related to ii‐CBCT acquisition is scattered X‐rays of MV beams from a patient (MV‐scatters), which are incident on the flat panel detector (FPD) of the linac‐mounted kV imaging subsystem. The authors in the above studies used a Catphan phantom (Phantom Laboratory, Salem, NY, USA) to evaluate the image quality of the ii‐CBCT images, which did not mimic human anatomy. Boylan et al. demonstrated correction methods for MV‐scatters using an anthropomorphic phantom and prostate VMAT patients.^13^ The authors subtracted MV‐scatter maps from the kV projections acquired during 15 MV beam delivery, as follows: (a) 2‐dimensional (2D) MV‐scatter maps of the phantom, (b) mean 2D maps of the phantom, (c) 2D maps of another phantom, and (d) maps estimated using an analytical model. It was revealed that subtracting the MV‐scatter map acquired by the same object was appropriate for the correction. However, only a qualitative visual evaluation was conducted for the patient ii‐CBCT images.

In recent years, in‐treatment magnetic resonance (MR) images acquired using MR‐cobalt or MR‐linac machines, which have been introduced in radiotherapy treatment, have provided superior soft‐tissue contrast over in‐treatment CBCT images.^15^ However, these machines are not commonly installed globally and cannot perform VMAT or non‐coplanar MV beam deliveries at present. From this perspective, ii‐CBCT acquisition is a preferable option because linacs with kV imaging subsystems are in widespread use.

The purpose of this study was (a) to investigate the basic characteristics of MV‐scatters relating to various MV beam parameters [field size (FS), MV energy, dose rate (DR), and gantry speed] on concurrent kV imaging projections, and (b) to evaluate the qualities of MV‐scatter–contaminated and MV‐scatter–corrected ii‐CBCT images using a pelvis phantom, which were acquired during 1‐arc or 2‐arc 15 MV prostate VMAT deliveries.

## 2 MATERIALS AND METHODS

We used the TrueBeam STx linac (Varian Medical Systems, Palo Alto, CA, USA) with a PaxScan 4030CB FPD (Varian Medical Systems), which has an active imaging area and matrix of 39.7 × 29.8 cm^2^ and 1,024 × 768 pixels, respectively. The Developer Mode option was used to perform concurrent kV imaging during MV beam irradiation, which is not allowed to use for human or animals. Concurrent kV imaging for a patient can be performed in the “Treatment Mode” on TrueBeam. However, the acquired images are recorded as a movie DICOM file and cannot be exported to the CBCT Reconstructor.

### 2.A Experiment 1: Effect of MV beam parameters on MV‐scatter

**Fig 1:**
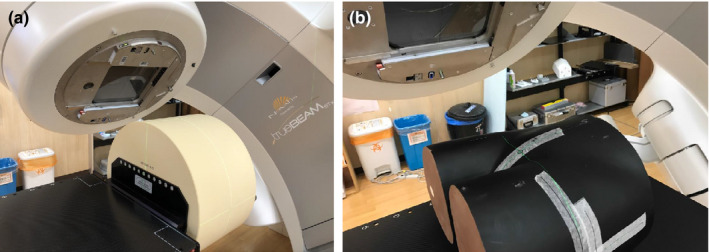
(a) Norm phantom and (b) pelvis phantom used in this study.

To evaluate the effects of the MV beam parameters on the MV‐scatter in the projections acquired during MV beam irradiation, concurrent kV imaging was performed during one full arc (360° rotation; clockwise) rotational delivery by changing the following MV beam parameters while the others remained fixed: FS, DR, and gantry speed. The kV imaging parameters and variations in the MV beam parameters are summarized in Tables 1 and 2, respectively. A cylindrical Norm phantom (40 cm diameter; Varian Medical Systems) was used, which was attached to the end of the patient couch [Fig. 1(a)].

**Table 1:** Concurrent kV imaging parameters for MV beam parameter variation experiments (Experiment 1).

kV imaging parameter	Description
Tube voltage [kV], tube current [mA], pulse time [ms]	125, 60, 20
Exposure per projection [mAs]	1.2
Filter, bowtie filter	Titanium, Half fan
kV blade position [cm]	X1 = 24.7, X2 = 3.3 and Y1 = Y2 = 10.7
Source‐to‐isocenter distance [cm]	100
Isocenter‐to‐detector distance [cm]	50
Detector offset [cm]	16
Frame rate [fps]	7

**Table 2:** Conditions for each MV beam parameter variation experiments (Experiment 1).

	Variation: Field size, MV energy	Variation: Dose rate	Variation: Gantry speed @ 100 MU/min	Variation: Gantry speed @ 300 MU/min
MV energy [MV]	6, 15	15	15	15
Field size [cm^2^]	3 × 3, 10 × 10, 15 × 15	10 × 10	10 × 10	10 × 10
Dose rate [MU/min]	600	100, 300, 600	100	300
Total monitor unit [MU]	600	100 for 100 MU/min, 300 for 300 MU/min, 600 for 600 MU/min	100, 300	300, 600
Gantry speed [°/s]	6	6	2 for 300 MU, 6 for 100 MU	3 for 600 MU, 6 for 300 MU
Gantry rotation angle [°]	360 (clockwise)	360 (clockwise)	360 (clockwise)	360 (clockwise)

For evaluation, 100 × 100 pixel regions of interest (ROIs) was set at the center of the MV‐scatter–contaminated projections and kV‐only projections at gantry angles of 0° to 315° with increments of 45°. The pixel values were averaged, and the standard deviations were calculated for the ROIs in each projection.

### 2.B Experiment 2: Concurrent kV imaging during VMAT delivery

#### 2.B.1 Plan characteristics and MV‐scatter correction

Eleven 1‐arc and seven 2‐arc 15 MV prostate VMAT plans were selected for the concurrent kV imaging experiment. At our institution, the 1‐arc plans were used routinely, whereas the 2‐arc plans were used if greater target coverage was necessary. The prescribed doses were typically 74 Gy in 37 fractions for the T1‐2b cases and 78 Gy in 39 fractions for the T2c cases.^16^.The target was local prostate only, lymph nodes were not irradiated. The plan details are described in Table 3. Each plan was created and optimized by the Eclipse treatment planning system (version 11.0, Varian Medical Systems). Moreover, each DICOM‐RT plan was exported and converted into XML files using the Veritas software (version 1.0, Varian Medical Systems). Control points for concurrent kV imaging were added to the files (Table 4). To compare the difference between MV energies, one plan was selected from each of 1‐arc and 2‐arc plans. MV energy in the xml files of selected plans was changed to 6 MV. A pelvis phantom (BrainLab, Munich, Germany) was irradiated and scanned, which was set up by matching the marking lines with a room laser in the head‐first spine position, without immobilization devices [Fig. 1(b)].

**Table 3:** Details of prostate volumetric‐modulated arc therapy plans used in this study (Experiment 2).

Plan information	Description
Number of plans	1‐arc: 11; 2‐arc: 7
MV beam delivery method	Volumetric‐modulated arc therapy with NDS120HD multi‐leaf collimator
MV beam energy	15 MV photon beam with flattening filter (15X‐FF)
Prescribed dose	74 Gy or 78 Gy in 2 Gy/fraction
Gantry rotation angle [°]	1‐arc: 181 – 179 (clockwise); 2‐arc: 181 – 179 (clockwise), 179 – 181 (counterclockwise)
Collimator angle [°]	Up to ±30 (depending on plan)
Median irradiated monitor unit per arc [MU]	1‐arc: 523.0 ± 45.6; 2‐arc: 272.9 ± 25.6

**Table 4:** Imaging parameters for concurrent kV imaging during prostate volumetric‐modulated arc therapy (Experiment 2).

kV imaging parameter	Description
Tube voltage [kV], tube current [mA], pulse time [ms]	125, 60, 20
Exposure per projection [mAs]	1.2
Filter, bowtie filter	Titanium, Half fan
kV blade position [cm]	*P*_MVkV_: X1 = 24.7, X2 = 3.3 and Y1 = Y2 = 10.7;
*P*_MVS_: X1 = X2 = 0 and Y1 = Y2 = 0	
Source‐to‐isocenter distance [cm]	100
Isocenter‐to‐detector distance [cm]	50
Detector offset [cm]	16
Frame rate [fps]	15
Reconstructed image slice thickness [mm]	2
Reconstructed image matrix [pixels]	512 × 512

Abbreviations: *P*_MVkV_: kV projection with open kV blades acquired during MV beam irradiation; *P*_MVS_: kV projection with closed kV blades acquired during MV beam irradiation, which contains MV‐scatter only.

**Fig 2:**
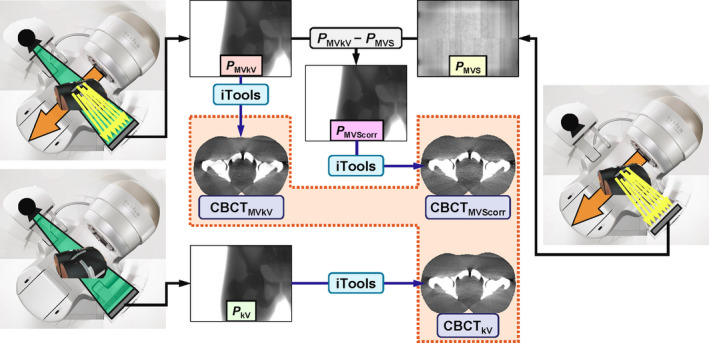
MV‐scatter correction scheme. CBCT_kV_: reconstructed CBCT image from kV projections consisting of kV beam only (*P*_kV_); CBCT_MVkV_: reconstructed CBCT image from kV projections consisting of kV beam and MV‐scatter (*P*_MVkV_);*P*_MVS_: projections consisting of MV‐scatter only;*P*_MVScorr_: MV‐scatter corrected projections; CBCT_MVScorr_: reconstructed CBCT image from*P*_MVScorr_.

The MV‐scatter correction method is summarized in Fig. 2. Firstly, the subject was imaged using a kV imaging subsystem during rotational MV beam irradiation (*P*_MVkV_). In this case, kV projections consisting of MV‐scatter only (*P*_MVS_) had to be generated to correct the MV‐scatters on *P*_MVkV_. Thus, concurrent kV imaging was conducted using closing kV blades, following which *P*_MVS_ was acquired. For 2‐arc plans, *P*_MVS_ were acquired by each arc. Thereafter, *P*_MVS_ was subtracted from the corresponding *P*_MVkV_ angle wise (*P*_MVScorr_) and the subtraction was demonstrated pixel wise. The correction could be expressed as:

(1) $$P_{\text{MVScorr}}\left(\theta \right)=P_{\text{MVkV}}\left(\theta \right)‐P_{\text{MVS}}\left(\theta \right)$$

(2) $$\sigma_{\text{MVScorr}}\left(\theta \right)=\sqrt{{\left\{\sigma_{\text{MVkV}}\left(\theta \right)\right\}}^{2}+{\left\{\sigma_{\text{MVS}}\left(\theta \right)\right\}}^{2}},$$

where σ and θ are the image noise and projection angle, respectively. Furthermore, the subject was scanned using a kV beam only (*P*_kV_).

The CBCT images were reconstructed from *P*_MVkV_, *P*_MVScorr_, and *P*_kV_ by iTools (Version 3.0, Varian Medical Systems) using the Feldkamp–Davis–Kress reconstruction algorithm (CBCT_MVkV_, CBCT_MVScorr_, and CBCT_kV_ as the reference image).^17^ iTools is an independent CBCT reconstruction software which has the same capability of CBCT Reconstructor system in TrueBeam linacs.

#### 2.B.2 Image quality evaluation

The root‐mean‐square error (RMSE) was calculated in prostate region for the ii‐CBCT images compared to CBCT_kV_ for the quantitative evaluation. A 50 × 50 × 30 pixels cuboid volume of interest (VOI; superior: top of femoral heads, inferior: middle of pubis; anterior: posterior pubic symphysis, posterior: anterior coccyx; right (left): inside right (left) ilium) was defined in lesser pelvis region. The RMSE of CBCT*_i_* (*i* = MVkV or MVScorr) was defined as

(3) $${\text{RMSE}}_{{\text{CBCT}}_{i}}=\sqrt{\frac{1}{N_{u}N_{v}N_{s}}\sum_{s=1}^{N_{s}}\sum_{u=1}^{N_{u}}\sum_{v=1}^{N_{v}}{\left\{{\text{HU}}_{{\text{CBCT}}_{i}}\left(u,v;s\right)‐{\text{HU}}_{{\text{CBCT}}_{\text{kV}}}\left(u,v;s\right)\right\}}^{2}},$$

where $\text{HU}\left(u,v;s\right)$ is the CT number at pixel position $\left(u,v\right)$ in slice *s*. Moreover, $N_{u}$ and $N_{v}$, and $N_{s}$ are the total number of pixels along the *u* and *v* directions, and the total number of slices, respectively.

Furthermore, 3D gamma analysis was applied for each ii‐CBCT compared to CBCT_kV_. VOI for the analysis was the entire range of the image, and different from the VOI in lesser pelvis region defined above. Low et al. introduced the original method for analyzing intensity‐modulated radiotherapy dose distributions.^18^, ^19^ CBCT‐numbers were used instead of doses. The CBCT‐number difference criterion was selected as 30 HU as it has been recommended that the CT number for materials with water‐like densities should be within 30 HU.^20^ The distance‐to‐agreement criterion was selected as 1 mm because the phantom was aligned by a room laser and rigidly registered. The pixel positions with CBCT‐numbers higher than −300 HU on CBCT_kV_, which was selected to avoid the patient couch being subject to analysis, were analyzed to calculate the gamma pass rates (GPRs) (30 HU/1 mm/−300 HU TH).

Moreover, subgroup analysis was performed for the GPRs related to the average multi‐leaf collimator (MLC) aperture and average DR during VMAT delivery. The MLC aperture for a control point was defined as the sum of the difference between the bank A and band B positions at each MLC pair. The total number of control points for the VMAT plans was 178 per arc.

## 3 RESULTS

### 3.A Experiment 1: Effect of MV beam parameters on MV‐scatter

**Fig 3:**
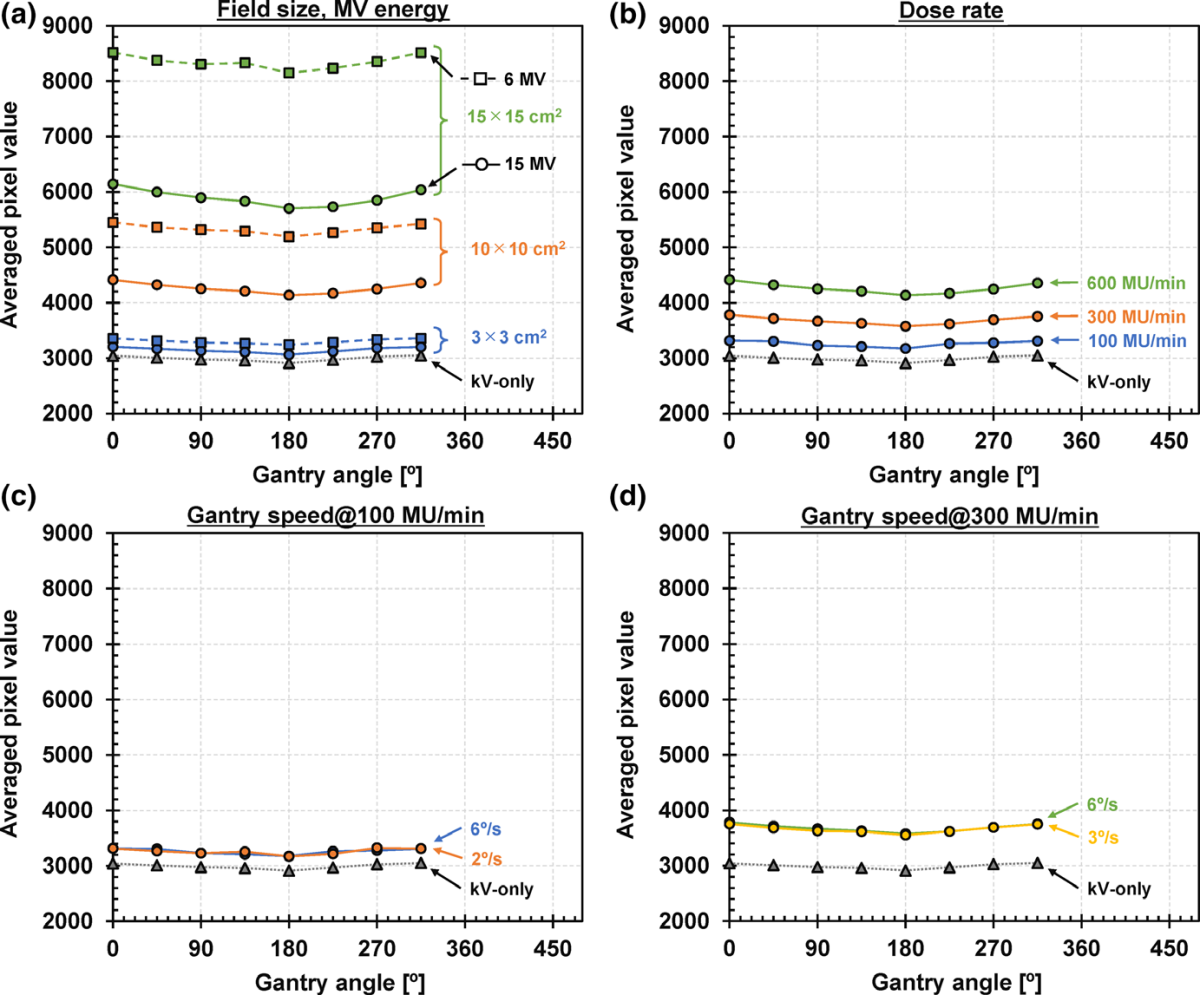
Result of the Experiment 1: MV‐scatter dependencies of (a) field size and MV energy, (b) dose rate, and gantry speed at dose rate of (c) 100 MU/min and (d) 300 MU/min.

The averaged pixel values at each gantry angle are presented in Fig. 3. Upon the results of the FS dependency [Fig. 3(a)], the average pixel values for the concurrent kV projections acquired during MV beam irradiation were increased compared to the kV‐only projections. The increases were due to MV‐scatters, and the MV‐scatters increased with larger FSs. In addition, the MV‐scatters increased with smaller MV energy which is 6 MV in this study. Moreover, the MV‐scatters increased with larger DRs. Contrariwise, the MV‐scatters did not depend on the gantry speed under the same DRs. The averaged pixel values at a gantry angle of 180° were relatively small as the starting position of the rotational delivery.

### 3.B Experiment 2: Concurrent kV imaging during VMAT delivery

**Fig 4:**
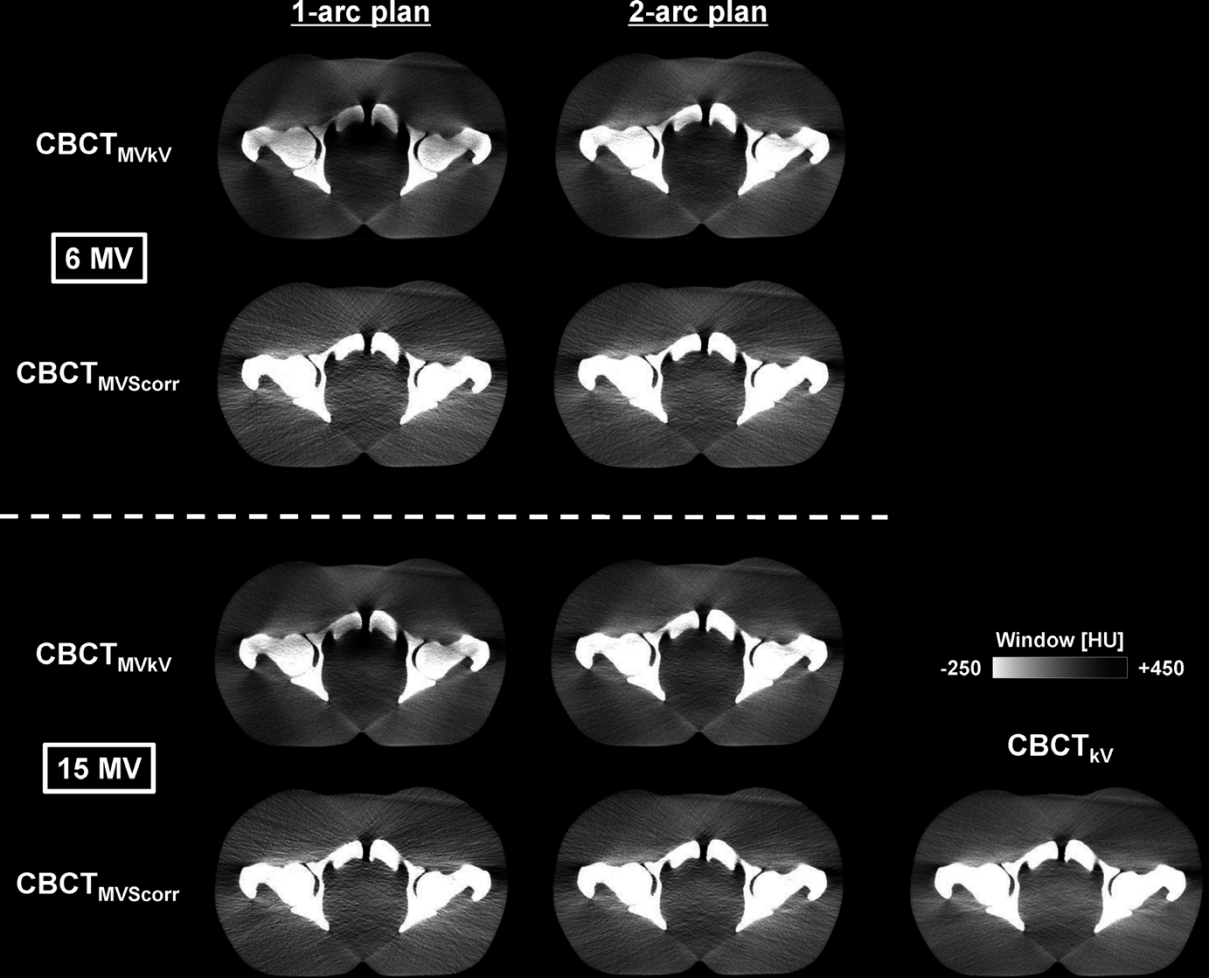
Intra‐irradiation CBCT images acquired during 6 and 15 MV volumetric‐modulated arc therapy deliveries for 1‐arc and 2‐arc plans and reference CBCT image. CBCT_MVkV_: reconstructed CBCT image from kV projections consisting of kV beam and MV‐scatter; CBCT_MVScorr_: reconstructed CBCT image from MV‐scatter corrected projections; CBCT_kV_: reconstructed CBCT image from kV projections consisting of kV beam only.

**Fig 5:**
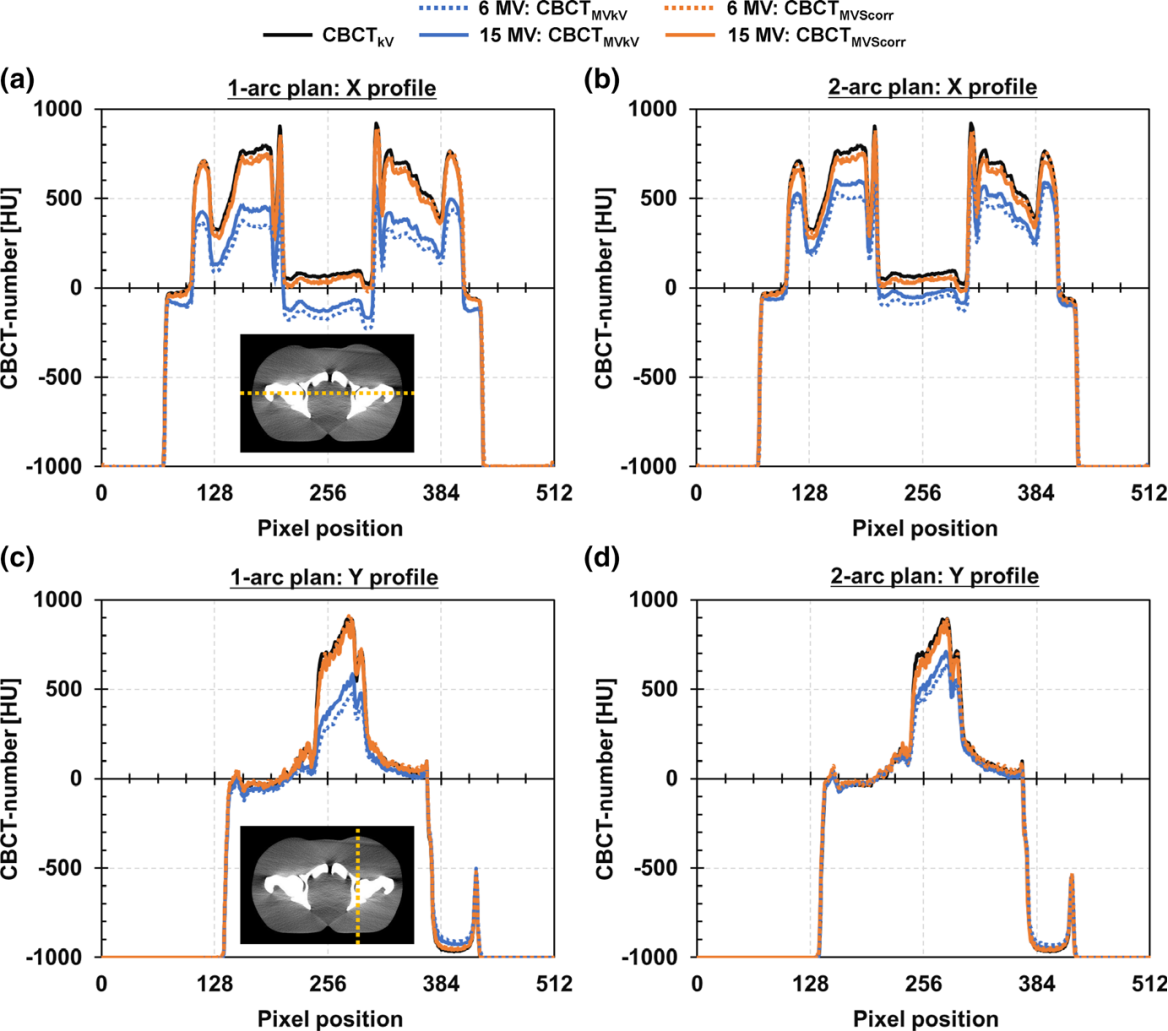
CBCT‐number profiles for 1‐arc plan along (a) X and (c) Y directions, and those for 2‐arc plan along (b) X and (d) Y directions on CBCT images acquired during 6 and 15 MV volumetric‐modulated arc therapy deliveries.

**Fig 6:**
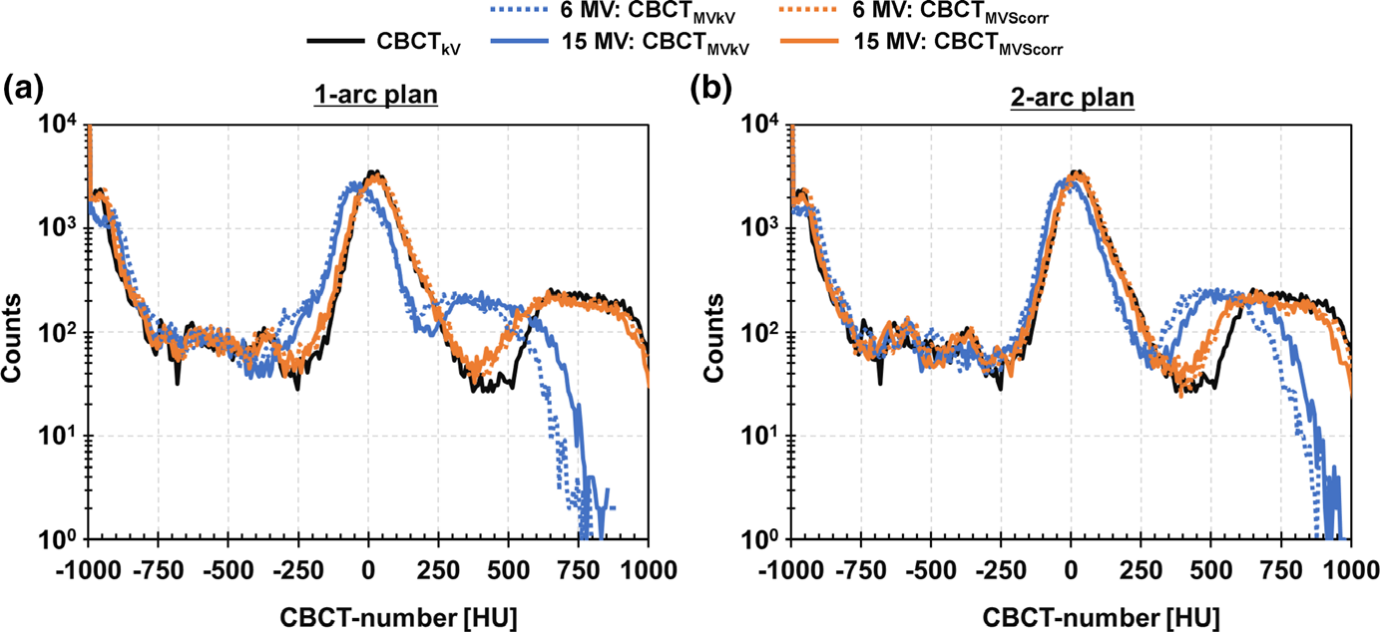
CBCT‐number histograms of CBCT images acquired during 6 and 15 MV volumetric‐modulated arc therapy deliveries for (a) 1‐arc and (b) 2‐arc plans.

The reference CBCT images (CBCT_kV_) and ii‐CBCT images (CBCT_MVkV_ and CBCT_MVScorr_) for one 1‐arc plan and one 2‐arc plan of 6 and 15 MV VMAT are presented in Fig. 4. In the qualitative evaluation, the posterior pubic symphysis and anterior femoral heads became dark for CBCT_MVkV_s compared to CBCT_kV_ owing to the cupping artifact. The artifact on the image acquired during 6 MV VMAT was larger than that during 15 MV VMAT. In contrast, both CBCT_MVScorr_s were comparable to CBCT_kV_. The CBCT‐number profiles along the X and Y directions, and CBCT‐number histograms are illustrated in Figs. 5 and 6, respectively. The effects of the cupping artifact (decrease in the CBCT‐numbers) on CBCT_MVkV_ of 6 MV VMAT were larger than those of 15 MV VMAT. Moreover, the effects of the cupping artifact on CBCT_MVkV_ of the 1‐arc plan were larger than those of the 2‐arc plan. The CBCT‐number profiles of both CBCT_MVScorr_s were comparable to those of CBCT_kV_.

**Fig 7:**
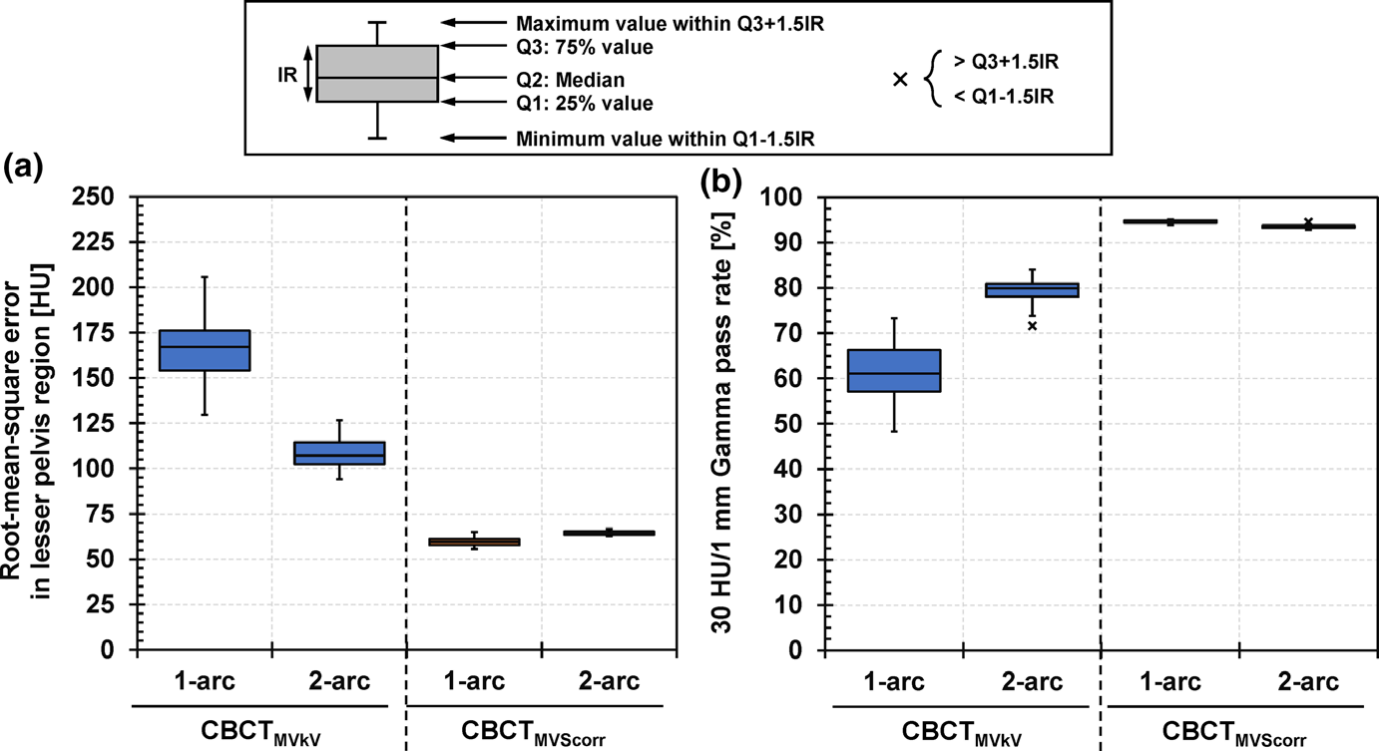
Boxplots of (a) root‐mean‐square error in lesser pelvis region and (b) 30 HU/1 mm Gamma pass rate. CBCT_MVkV_: reconstructed CBCT image from kV projections consisting of kV beam and MV‐scatter; CBCT_MVScorr_: reconstructed CBCT image from MV‐scatter–corrected projections.

**Fig 8:**
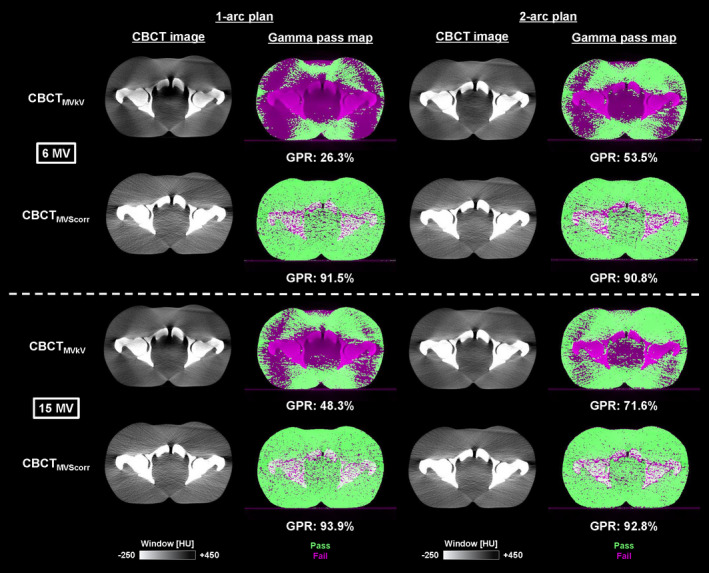
Intra‐irradiation CBCT images and gamma pass map for 1‐arc and 2‐arc plans acquired during 6 and 15 MV volumetric‐modulated arc therapy delivery. CBCT_MVkV_: reconstructed CBCT image from kV projections consisting of kV beam and MV‐scatter; CBCT_MVScorr_: reconstructed CBCT image from MV‐scatter–corrected projections; GPR: Gamma pass rate.

**Fig 9:**
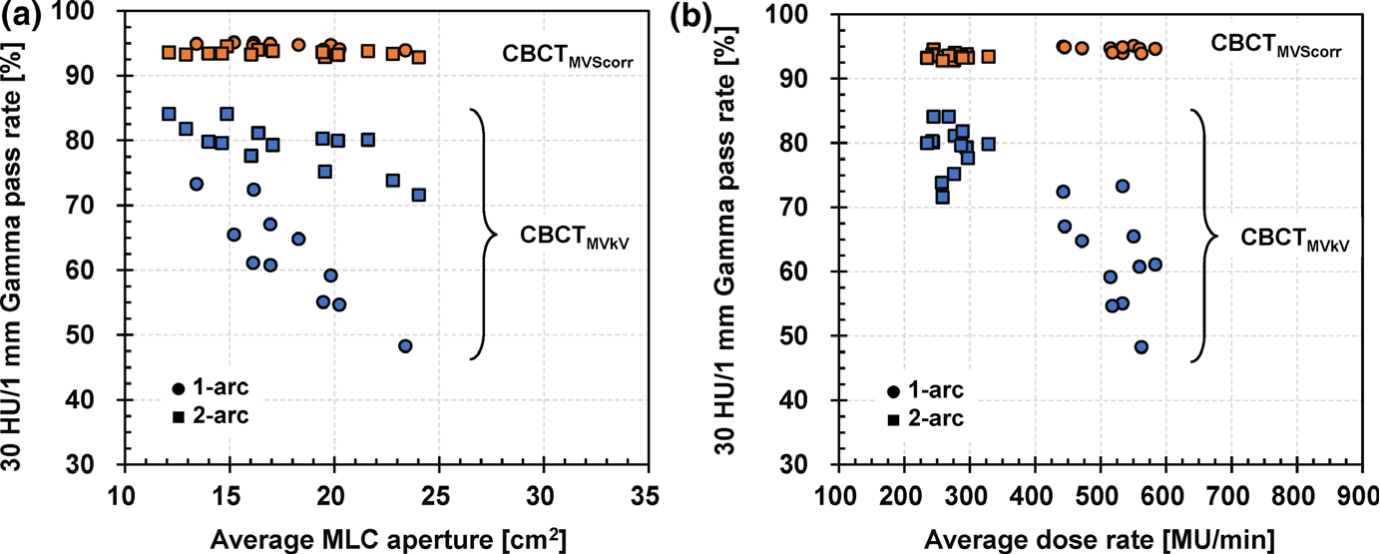
Subgroup analysis of gamma pass rates relating to (a) average multi‐leaf collimator (MLC) aperture and (b) average dose rate. CBCT_MVkV_: reconstructed CBCT image from kV projections consisting of kV beam and MV‐scatter; CBCT_MVScorr_: reconstructed CBCT image from MV‐scatter–corrected projections.

The boxplots of the RMSE and 30 HU/1 mm/−300 HU TH 3D GPR in the quantitative analysis for eleven 1‐arc and seven 2‐arc 15 MV prostate VMAT plans are presented in Fig. 7. The median RMSEs of CBCT_MVkV_s for the 1‐arc and 2‐arc plans were 167.0 and 107.2 HU, respectively. The values of CBCT_MVScorr_s were reduced to 59.57 and 64.37 HU, respectively. The median GPRs of CBCT_MVkV_s for the 1‐arc and 2‐arc plans were 61.1% and 79.9%, respectively. Those of CBCT_MVScorr_s increased to 94.7% and 93.4%, respectively. The CBCT images and gamma pass maps for the 1‐arc and 2‐arc plans, indicating the minimum GPRs in both arc plans, are illustrated in Fig. 8. In the gamma pass map, the failure positions were distributed around the pelvic bone, and the center and lateral peripheral regions of the phantom. Moreover, subgroup analyses relating to the average MLC apertures and average DRs during the VMAT deliveries were conducted (Fig. 9). The average DR exhibited a stronger negative correlation with the GPRs than the average MLC aperture, with a correlation coefficient of −0.86 for the former and −0.43 for the latter.

The difference of GPR between 6 MV and 15 MV VMAT is shown in Fig. 8. GPRs of CBCT_MVkV_s acquired during 6 MV VMAT for the 1‐arc and 2‐arc plans were 26.3% and 53.5%, while those acquired during 15 MV VMAT were 48.3% and 71.6%, respectively. The image quality of CBCT_MVkV_ acquired during 6 MV VMAT was degraded compared to that acquired during 15 MV VMAT since more MV‐scatters were generated, which was supported by the results of the Experiment 1 [Fig. 3(a)].

## 4 DISCUSSION

In this study, the effects of the MV beam parameters on MV‐scatters were investigated (Experiment 1) and quality evaluations of ii‐CBCT images acquired during 1‐arc and 2‐arc prostate VMAT deliveries were conducted (Experiment 2). The MV‐scatters were side‐scattered X‐rays as a result of Compton scattering.^21^ Thus, the number of MV‐scatters at a certain time was proportional to the number of irradiated MV beam photons. As the FSs and DRs were the dominant parameters for the number of MV beam photons, the averaged pixel values (number of MV‐scatters) increased with larger parameters. In addition, according to the Compton scattering, low‐energy X‐ray will be largely side scattered. Thus, the MV‐scatters increased with 6 MV compared to 15 MV, which was supported by Experiment 1 [Fig. 3(a)]. Moreover, the GPRs decreased with larger FSs and DRs, and smaller MV energy, as illustrated in Fig. 9.

As indicated in Fig. 3, the concurrent kV projections, or *P*_MVkV_, consisted of primary kV photons and MV‐scatters. Therefore, the pixel values increased compared to those of the kV‐only projections. This caused a beam‐hardening artifact on the reconstructed CBCT images. The effect of the artifact was pronounced in the center of the images and bone regions, as supported by Figs. 4, 5, 6, 8. The MV‐scatters were corrected in the projection domain by the MV‐scatter correction method, resulting in the reconstruction of appropriate CBCT‐numbers, with >90% GPRs.

As the highest priority during the VMAT deliveries for the TrueBeam machines was to achieve a gantry speed of 6°/s, the irradiation time for one arc was almost the same regardless of whether the 1‐arc or 2‐arc plans, which was approximately 1 rpm. Thus, the MUs for each arc in the 2‐arc plans were optimized to be smaller than that for the one arc in the 1‐arc plan, resulting in smaller average DRs during the VMAT deliveries. Therefore, the effect of the MV‐scatters on the ii‐CBCT images for the 2‐arc plans was small compared to that for the 1‐arc plans. This trend can be observed in the results of the subgroup analysis of the GPRs [Fig. 9(b)]. Although the average MLC apertures did not differ between the 1‐arc and 2‐arc plans [Fig. 9(a)], the results showed two clusters because the average DRs differed.

The improvements in the median RMSEs of CBCT_MVScorr_ from CBCT_MVkV_ for the 2‐arc plan were slightly smaller than those for the 1‐arc plan, although the number of MV‐scatters for the 2‐arc plan was smaller than that of the 1‐arc plan, as discussed above. However, the GPRs of CBCT_MVScorr_ for the 2‐arc plans, which considered the CBCT‐number difference and distance‐to‐agreement simultaneously, were comparable with those for the 1‐arc plan.

The demonstrated MV‐scatter correction method required the measured *P*_MVS_. However, this requirement may be an obstacle from a clinical perspective, as the measurement of patient‐specific *P*_MVS_ prevents ii‐CBCT acquisition at the first fraction. It may be a more critical issue in hypofractionated treatment, such as 42.7 Gy in seven fractions for prostate cancer.^22^ There are several methods to solve this issue, as follows: (a) Measuring *P*_MVS_ by irradiating an individual VMAT plan to an anthropomorphic phantom such as CTU‐41 (Kyoto Kagaku Co., Ltd, Kyoto, Japan), which mimics human organs. (b) Estimating *P*_MVS_ using MV‐scatter database. Iramina et al. comprehensively measured the MV‐scatter by varying the MV beam parameters and built an MV‐scatter database.^23^ And the author proposed and demonstrated that MV‐scatters can be corrected by estimated *P*_MVS_s from certain MV beam parameters. (c) Estimating *P*_MVS_ using Monte Carlo simulation, whereby the patient‐specific *P*_MVS_ can be generated by inputting an individual planning CT image to a dedicated Monte Carlo geometry for concurrent kV imaging.

When VMAT deliveries are used, ii‐CBCT images can be acquired in various regions, such as the head and neck (H&N), thorax, abdomen, pelvis, and prostate. For the H&N and thoracic regions, low‐MV energy beams such as 6 or 8 MV are often used. As mentioned above, MV‐scatters increase with lower MV energies,^21^ which may degrade the image quality of the ii‐CBCT images compared to larger MV energy such as 10 or 15 MV. For abdominal treatment, such as stereotactic body radiotherapy for the liver or pancreas, high‐DR flattening filter‐free beams are often used, which generate more MV‐scatters compared to flattening filter beams of the same MV energy since the DR was the dominant parameters for the number of MV beam photons. For the pelvic region, the average MLC apertures may be large compared to those of localized prostate treatment. In addition, the scatterer volume, or patient volume in clinical practice, is another dependency for *P*_MVS_. The MV‐scatters may increase with larger scatterer volume. Patient weight loss during radiotherapy is often occurred if the number of fractions is large. In this case, measured *P*_MVS_ acquired at the first fraction, or estimated ones, may not be appropriate at the near end of the treatment. The effectiveness of MV‐scatter correction on ii‐CBCT images for other regions and the scatterer volume dependency for *P*_MVS_ should be investigated in future studies.

## 5 CONCLUSIONS

In this study, the effects of MV‐scatters were evaluated by varying the MV beam parameters. Moreover, to the best of our knowledge, this is the first study to evaluate the image quality of ii‐CBCT images of 1‐arc and 2‐arc prostate VMAT deliveries quantitatively using the RMSE and 3D gamma analysis. The number of MV‐scatters increased with larger FSs, higher DRs, and smaller MV energy. Although the effects of the MV‐scatters resulted in cupping artifacts on the ii‐CBCT images, the MV‐scatters were corrected in the MV‐scatter–corrected ii‐CBCT images by MV‐scatter correction, which exhibited >90% GPRs regardless of whether the 1‐arc or 2‐arc plan was used.

## CONFLICT OF INTEREST

The authors of this publication have no conflict of interest to declare.
